# G9α‐dependent histone H3K9me3 hypomethylation promotes overexpression of cardiomyogenesis‐related genes in foetal mice

**DOI:** 10.1111/jcmm.14824

**Published:** 2019-11-19

**Authors:** Bohui Peng, Xiao Han, Chang Peng, Xiaomei Luo, Ling Deng, Lixin Huang

**Affiliations:** ^1^ Department of Pediatrics Affiliated Hospital of Zunyi Medical University Zunyi China; ^2^ Department of Physiology School of Basic Medical Sciences Zunyi Medical University Zunyi China

**Keywords:** alcohol consumption, cardiomyogenesis, histone methylation, mice, pregnancy

## Abstract

Alcohol consumption during pregnancy can cause foetal alcohol syndrome and congenital heart disease. Nonetheless, the underlying mechanism of alcohol‐induced cardiac dysplasia remains unknown. We previously reported that alcohol exposure during pregnancy can cause abnormal expression of cardiomyogenesis‐related genes, and histone H3K9me3 hypomethylation was observed in alcohol‐treated foetal mouse heart. Hence, an imbalance in histone methylation may be involved in alcohol‐induced cardiac dysplasia. In this study, we investigated the involvement of G9α histone methyltransferase in alcohol‐induced cardiac dysplasia in vivo and in vitro using heart tissues of foetal mice and primary cardiomyocytes of neonatal mice. Western blotting revealed that alcohol caused histone H3K9me3 hypomethylation by altering G9α histone methyltransferase expression in cardiomyocytes. Moreover, overexpression of cardiomyogenesis‐related genes (*MEF2C*, *Cx43*, *ANP* and *β‐MHC*) was observed in alcohol‐exposed foetal mouse heart. Additionally, we demonstrated that G9α histone methyltransferase directly interacted with histone H3K9me3 and altered its methylation. Notably, alcohol did not down‐regulate H3K9me3 methylation after G9α suppression by short hairpin RNA in primary mouse cardiomyocytes, preventing MEF2C, Cx43, ANP and β‐MHC overexpression. These findings suggest that G9α histone methyltransferase‐mediated imbalance in histone H3K9me3 methylation plays a critical role in alcohol‐induced abnormal expression cardiomyogenesis‐related genes during pregnancy. Therefore, G9α histone methyltransferase may be an intervention target for congenital heart disease.

## 1 INTRODUCTION

Congenital heart disease (CHD) is one of the most common congenital malformations, consisting of abnormalities in the structure and function of the newborn's cardiovascular system. Congenital heart disease cases are estimated to range from 8 to 10 per 1000 live births.^1^, ^2^, ^3^ Cardiac development is a very intricate process regulated by precise temporal and spatial expression patterns of heart development‐related genes. Several studies have shown that both genetic and epigenetic factors play a critical role in the expression of cardiomyogenesis genes.^4^, ^5^, ^6^ As a common environmental teratogen, alcohol consumption during pregnancy has been reported to cause CHD in foetuses.^7^, ^8^ Unfortunately, the underlying mechanism remains unclear. Although the events responsible for cardiac development are not known in detail, increasing evidence supports the involvement of altered histone methylation in the regulation of cardiomyogenesis.^9^, ^10^ Histone methylation includes monomethylation, dimethylation and trimethylation. Methylation affects the transcriptional activity of cardiac nuclear transcription factors and, therefore, the activation or repression of downstream cardiomyogenesis‐related genes.

G9α histone methyltransferase (G9α‐HMT) plays a dominant role in euchromatic histone H3 lysine 9 methylation. Some studies have shown that euchromatic H3K9 methylation regulated by G9α is involved in the transcriptional repression of developmental genes.^11^ In our previous studies, we demonstrated that alcohol consumption during pregnancy induces hyperacetylation of histone H3K9ac and causes overexpression of cardiomyogenesis genes.^12^, ^13^ However, the inhibition of histone H3K9ac hyperacetylation did not completely reverse heart malformation caused by alcohol consumption during pregnancy. Recently, we demonstrated that the interactive control of histone acetylation and methylation is critical for heart development.^14^ Therefore, we hypothesized that the imbalance in histone methylation may be involved in alcohol‐induced cardiac dysplasia. In the present study, we verified this hypothesis by investigating the effects of G9α‐HMT on histone H3K9me3 and on the expression of cardiomyogenesis genes in alcohol‐exposed foetal mouse hearts. This study may provide new intervention targets for the prevention and treatment of alcohol‐induced CHD.

## 2 MATERIALS AND METHODS

### 2.1 Experimental mice

Pathogen‐free Kunming mice (10‐12 weeks old, both male and female) with a body mass of 25‐30 g were purchased from the Experimental Animal Center at Chongqing Medical University (Chongqing, China). All procedures on experimental animals were performed in compliance with relevant laws and institutional guidelines and were approved by the Animal Care and Use Committee of Zunyi Medical University. Mice were maintained under fully controlled conditions (22 ± 1°C, 55% ± 5% humidity) with a 12‐hour light:12‐hour dark cycle and were allowed ad libitum food access. After they had mated in the evening (19:00), the presence of vaginal plug was verified in the mated females at 8:00 on the following morning. If a vaginal plug was observed, embryos were considered to be at embryonic day 0.5 (ED 0.5). At 8:00 every morning, pregnant mice were gavaged with 5 mL/kg of 56% ethanol (v/v) from ED 7.5 to ED 15.5. Control mice received an equivalent volume of normal saline. Pregnant mice were euthanized using carbon dioxide asphyxia, and the embryonic hearts were promptly collected from mouse pups for further analyses.

### 2.2 Cell culture

Neonatal mouse ventricular myocytes from the hearts of 1‐ to 3‐day‐old Kunming mice were isolated under aseptic conditions. Briefly, mice were sacrificed by decapitation, and the hearts were obtained immediately and kept in cold phosphate‐buffered saline (PBS) for washing. Subsequently, the cardiac ventricles were minced using fine scissors and were cut into pieces of approximately 1‐2 mm^3^. The cardiomyocytes were dissociated by trituration in 0.05% collagenase type II (Worthington) 8‐10 times for 5 minutes each time. The cells were centrifuged and resuspended in DMEM/F12 (1:1) containing 20% foetal bovine serum (Invitrogen) after discarding the supernatant. Subsequently, the cells were incubated in humidified air with 5% CO_2_ at 37°C for 1 hour to separate the fibroblasts.

### 2.3 Short hairpin RNA (shRNA) and retroviral infections

One hundred nanograms of G9α‐specific shRNA was used. The lentivirus promoter driving the expression of shRNA and the shRNA sequence was inserted. The expression of the reporter, enhanced green fluorescent protein (eGFP), was driven by the Ubi promoter. shRNA and eGFP sequences were incorporated into a lentivirus. Lentiviruses were produced in 293T cells, and viral titres of 2 × 10^9^ TU/mL were used. The cells were seeded on a 6‐well culture plate at 1 day before infection. Fresh medium containing 5 µg/mL polybrene and the virus (MOI = 4) were added to the cells. Fluorescence signals were observed under a fluorescence microscope after 72 hours of cell infection.

### 2.4 Total RNA extraction and real‐time quantitative polymerase chain reaction (RT‐PCR)

Total RNA from myocardium cells was extracted with an RNA extraction kit (BioTeke). Single‐stranded cDNA was synthesized from 500 to 1000 ng of RNA using oligo dT‐adaptor primers and an AMV reverse transcriptase kit (Takara) following the manufacturer's instructions. Subsequently, cDNA was amplified with gene‐specific primers and an SYBR Green dye kit (Takara). β‐actin was used as internal reference. The Ct value in 2^−ΔΔCt^ indicated the relative gene expression.

### 2.5 Western blotting

Nucleoproteins were extracted using a nuclear extraction kit (Merck Millipore), separated, subjected to SDS‐PAGE and then transferred to polyvinylidene difluoride membranes using a Bio‐Rad semidry electrotransfer apparatus. The nitrocellulose membranes were blocked with 5% non‐fat milk in Tris‐buffered saline and incubated with monoclonal antibodies (anti‐G9α, anti‐H3K9me3, anti‐MEF2C, anti‐Cx43, anti‐ANP, anti‐β‐MHC and anti‐GAPDH [Abcam]) diluted in Tris‐buffered saline. Protein bands on immunoblots were visualized by enhanced chemiluminescence. After scanning, bands were quantified using Quantity One software version 4.4 (Bio‐Rad).

### 2.6 Immunofluorescence

Cardiomyocytes were detected by immunofluorescence. Myocardial cells were cultured on Thermanox plastic coverslips (Thermo Fisher Scientific), washed with PBS and fixed with 4% paraformaldehyde. The cells were treated with 0.1% Triton X‐100 in PBS and 10% horse serum and were subsequently incubated with primary antibody (anti‐G9α, anti‐H3K9me3 [1:200, Abcam]) at room temperature for 1 hour. After washing, Alexa Fluor 594 goat anti‐mouse IgG secondary antibody (1:1000, Thermo Fisher Scientific) was added and incubated in the dark at room temperature for 1 hour. Cells were washed with PBS and counterstained with DAPI before observation under a fluorescence microscope. All images were taken using the same microscope parameters, and fluorescence quantification on the images was performed using ImageJ software.^15^

### 2.7 Chromatin immunoprecipitation (ChIP)

ChIP assay was performed using a ChIP assay kit (Merck Millipore). After homogenization of the heart tissues, formaldehyde (1%) was added to the samples to cross‐link the DNA‐protein complexes. Immunoprecipitation was performed overnight by using specific antibodies (anti‐RNA polymerase II antibody) and normal mouse IgG as a negative control. After immunoprecipitation, NaCl was utilized to reverse the DNA‐protein crosslinks, and DNA was purified. Specific primers were designed for the promoters of *MEF2C, ANP*, *β‐MHC*, *Cx43* and *α‐actin* for RT‐PCR.

### 2.8 Co‐immunoprecipitation (CoIP)

Primary myocardial cells were subjected to immunoprecipitation and Western blotting procedures using primary anti‐G9α, anti‐H3K9ac and anti‐H3K9me3 rabbit polyclonal antibodies and Dynabeads Protein G (Invitrogen). The primary antibody was bound to the protein G magnetic beads according to manufacturer's instructions, and the target antigen (G9α) was immunoprecipitated in an immunoprecipitation buffer containing 1% Triton X‐100, 0.5% NP‐40, 20 mmol/L HEPES, 50 mmol/L NaCl and protease inhibitors, at pH 7.4, using a magnet. Subsequently, the samples were washed three times with lysis buffer. Immobilized protein complexes were eluted and denatured in 2× SDS sample buffer at 95°C for 10 minutes and were subsequently analysed by Western blotting with anti‐G9α and anti‐H3K9me3 antibodies, as described in Section 2.5. IgG was used as a negative control. The G9α immunoprecipitation experiments were performed in triplicate.

### 2.9 Statistical analysis

SPSS statistical software package version 18.0 (SPSS Inc, Chicago, IL, USA) was used for statistical analysis. All data are expressed as mean ± SD. Statistical analysis was performed using *t* test or one‐way ANOVA *P*‐values < .05 were considered statistically significant.

## 3 RESULTS

### 3.1 Effects of alcohol exposure on HMT activity and histone H3K9me3 methylation in mouse cardiomyocytes

**Figure 1:**
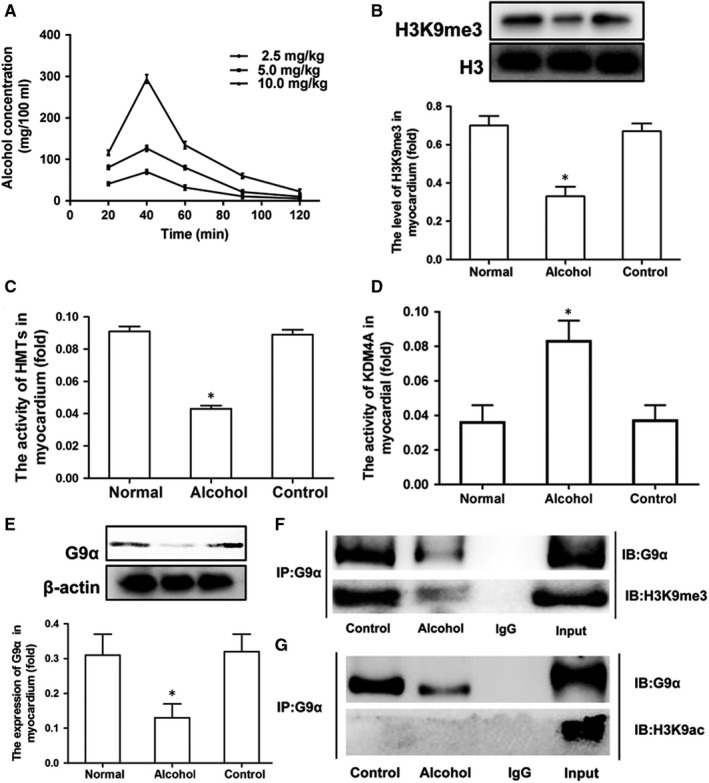
Effects of alcohol exposure on HMT activity and histone H3K9me3 hypomethylation. To analyse the impact of alcohol on HMT activity, different doses of alcohol were tested to identify optimal exposure conditions in the foetal mouse heart. (A) Blood alcohol concentration after gavaging with different doses of 56% ethanol in mice (n = 6). (B) A representative Western blotting shows that the methylation level of histone H3K9me3 was significantly decreased after treatment with alcohol. (C) Alcohol (56%) decreased HMT activity. (D) Alcohol (56%) increased KDM4A activity. (E) G9α‐HMT was significantly decreased in the mouse heart upon alcohol exposure. (F) Co‐immunoprecipitation (CoIP) in cell lysates of mouse myocardial cells exposed to two different experimental conditions with anti‐G9α‐protein G magnetic beads and immunoblot (IB) with an anti‐H3K9me3 or anti‐G9α antibody for evaluation of protein expression. Alcohol caused a dramatic decrease in the intensity of the G9α and H3K9me3 bands. (G) H3K9ac was used as a negative control. CoIP showed that G9α did not interact with H3K9ac in mouse myocardial cells. Input: positive control, IgG: negative control. *P < .05 vs the control group (n = 6)

We first ascertained the optimal alcohol dose to evaluate the effects on HMT activity. To this end, different volumes of 56% (v/v) ethanol in water were intragastrically administered to pregnant mice. The alcohol dose of 5 mL/kg was selected based on the blood alcohol concentration (Figure 1A). Western blotting analysis showed that the methylation level of histone H3K9me3 was significantly lower in the alcohol‐treated group than in the control group (*P* < .05; Figure 1B). The data indicated that alcohol significantly decreased HMT activity in myocardial tissues of foetal mice (*P* < .05; Figure 1C). Colorimetric assays showed that alcohol significantly increased KDM4A activity in myocardial tissues of foetal mice (*P* < .05; Figure 1D). Moreover, alcohol caused significant G9α‐HMT down‐regulation (*P* < .05; Figure 1E). CoIP experiments were conducted to verify the formation of a complex between G9α and H3K9me3 and demonstrated the occurrence of this interaction in neonatal myocardial cells. These data indicated that G9α might directly interact with H3K9me3 (Figure 1F). The CoIP experiments showed that G9α did not interact with H3K9ac in mouse myocardial cells (Figure 1G).

### 3.2 Alcohol promotes overexpression of cardiomyogenesis‐related genes in the hearts of foetal mice

**Figure 2:**
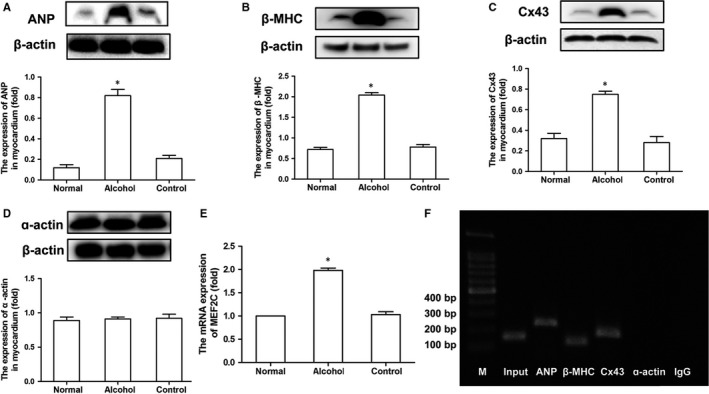
Effect of alcohol on protein expression of ANP, β‐MHC, and Cx43 and *MEF2C* transcription. (A, B, and C) Western blotting shows that the expression of the cardiomyogenesis gene atrial natriuretic peptide (*ANP*), beta‐myosin heavy chain (*β‐MHC*) and connexin 43 (*Cx43*) was significantly higher in alcohol‐treated mice than in control mice, whereas (D) α‐actin expression was unaffected. (E) qRT‐PCR shows that alcohol significantly increased *MEF2C* mRNA expression in mice. (F) ChIP‐PCR demonstrates that MEF2C bound to the promoters of *ANP*, *β‐MHC* and *Cx43*, but not to that of *α‐actin*. Input: positive control, IgG: negative control. *P < .05 vs the control group (n = 6)

The protein expression of ANP, β‐MHC, Cx43 and α‐actin was analysed by Western blotting. Alcohol significantly increased the expression of ANP, β‐MHC, and Cx43, but not that of α‐actin, compared to that in control mice (*P < *.05; Figure 2A‐D). The mRNA expression of *MEF2C*, encoding a critical transcription factor involved in heart development and many cardiovascular diseases, was tested by RT‐PCR *MEF2C* expression was higher in the alcohol‐treated than in the control group (*P < *.05; Figure 2E). In addition, we explored the relationship between *MEF2C* and downstream genes involved in cardiac development (*ANP*, *β‐MHC, α‐actin* and *Cx43*). The binding affinity of *MEF2C* for the promoters of *ANP*, *β‐MHC*, *Cx43* and *α‐actin* was examined by ChIP followed by PCR. We found that MEF2C could bind to the promoters of *ANP*, *β‐MHC* and *Cx43* but not to that of *α‐actin* (Figure 2F). The above results indicated that the heart nuclear transcription factor *MEF2C* could regulate the expression of cardiomyogenesis‐related genes.

### 3.3 Optimization of conditions for alcohol exposure and lentivirus‐mediated G9α knock‐down in primary mouse cardiomyocytes

**Figure 3:**
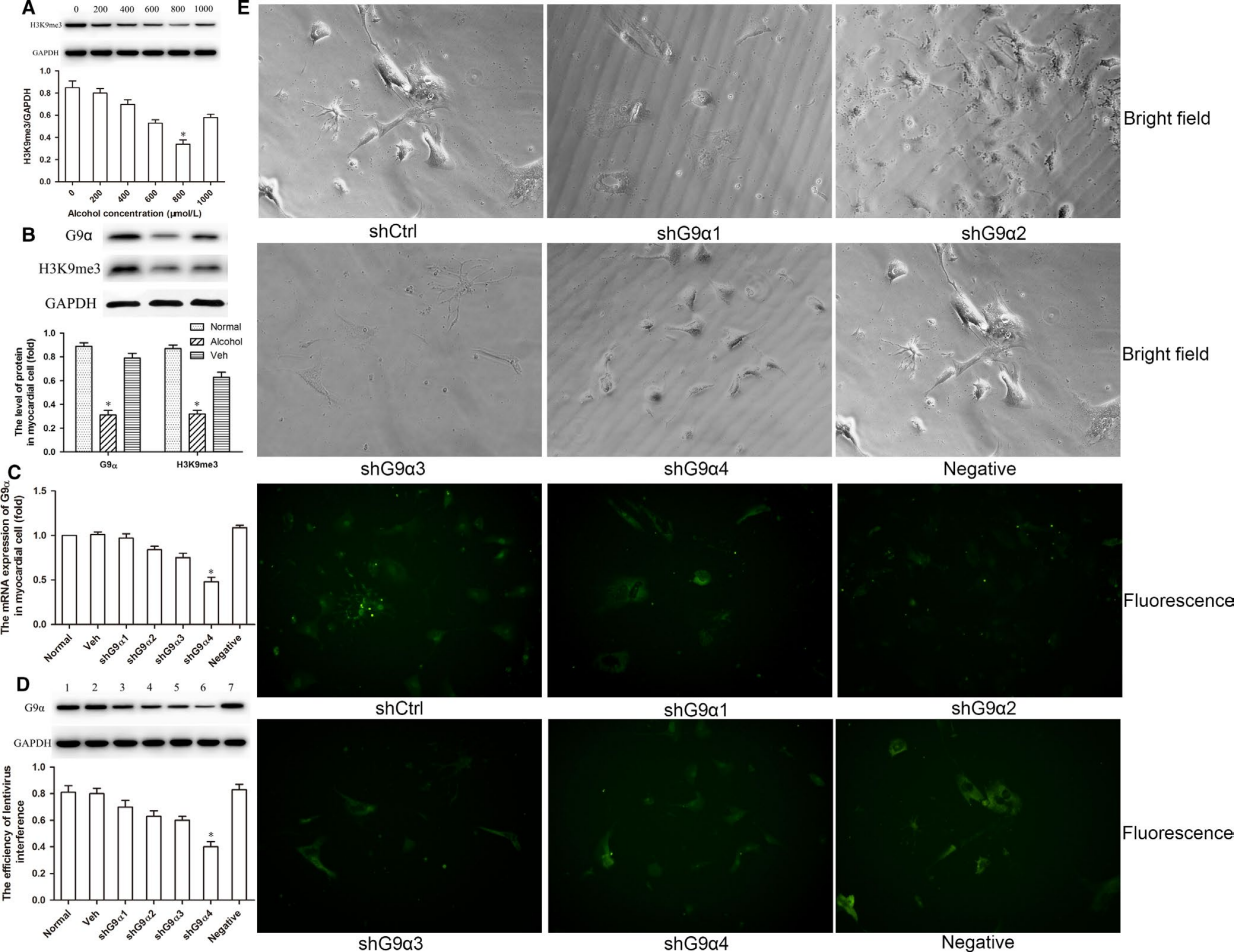
Lentivirus‐mediated shRNA transfection can efficiently and specifically knock‐down G9α gene expression in myocardial cells. (A) Different alcohol concentrations were used to determine the optimal exposure dose in primary cardiomyocytes of neonatal mice, and 800 µmol/L was selected based on the methylation level of H3K9me3. (B) The level of G9α and H3K9me3 showed a significant decrease in myocardial cells. (C) Four shG9α intervention sites were used to optimize the transfection efficiency, and shG9α4 was selected based on G9α mRNA expression in myocardial cells. (D and E) The efficiency of transfection with lentiviral vector containing G9α shRNA was analysed by Western blotting and immunofluorescence, respectively. The scale bars represent 50 μm. *P < .05 vs the control group (n = 6)

We first determined the optimal alcohol concentration in primary cardiac myocytes of neonatal mice. Western blotting was used to evaluate the expression of H3K9me3 in myocardial cells exposed to different alcohol concentrations. The lowest level of H3K9me3 expression was observed following cell exposure to 800 μmol/L alcohol (Figure 3A). The protein expression of both G9α and H3K9me3 was significantly decreased by alcohol exposure (*P* < .05 compared to control mice; Figure 3B). Moreover, in order to optimize lentivirus‐mediated G9α knock‐down, we first used alternative shRNA constructs (shG9α1, shG9α2, shG9α3 and shG9α4) targeting distinct G9α sites in cultured primary myocardial cells in vitro, followed by assessment of G9α mRNA and protein levels by RT‐PCR and Western blotting. Of the four constructs, shG9α4 resulted in the lowest level of shRNA expression (Figure 3C), and Western blotting showed that shG9α4 most strongly inhibited G9α protein expression (Figure 3D). Consistently, a higher number of shG9α4‐positive cells were detected by immunofluorescence, reflecting high transfection efficiency and the optimal targets of G9α were verified (Figure 3E). These data indicated that shG9α4 displayed the highest interference efficiency; thus, shG9α4 was selected for G9α knock‐down in myocardial cells of neonatal mice.

### 3.4 Lentiviral shRNA strongly decreases G9α expression and causes H3K9me3 hypermethylation in primary myocardial cells

**Figure 4:**
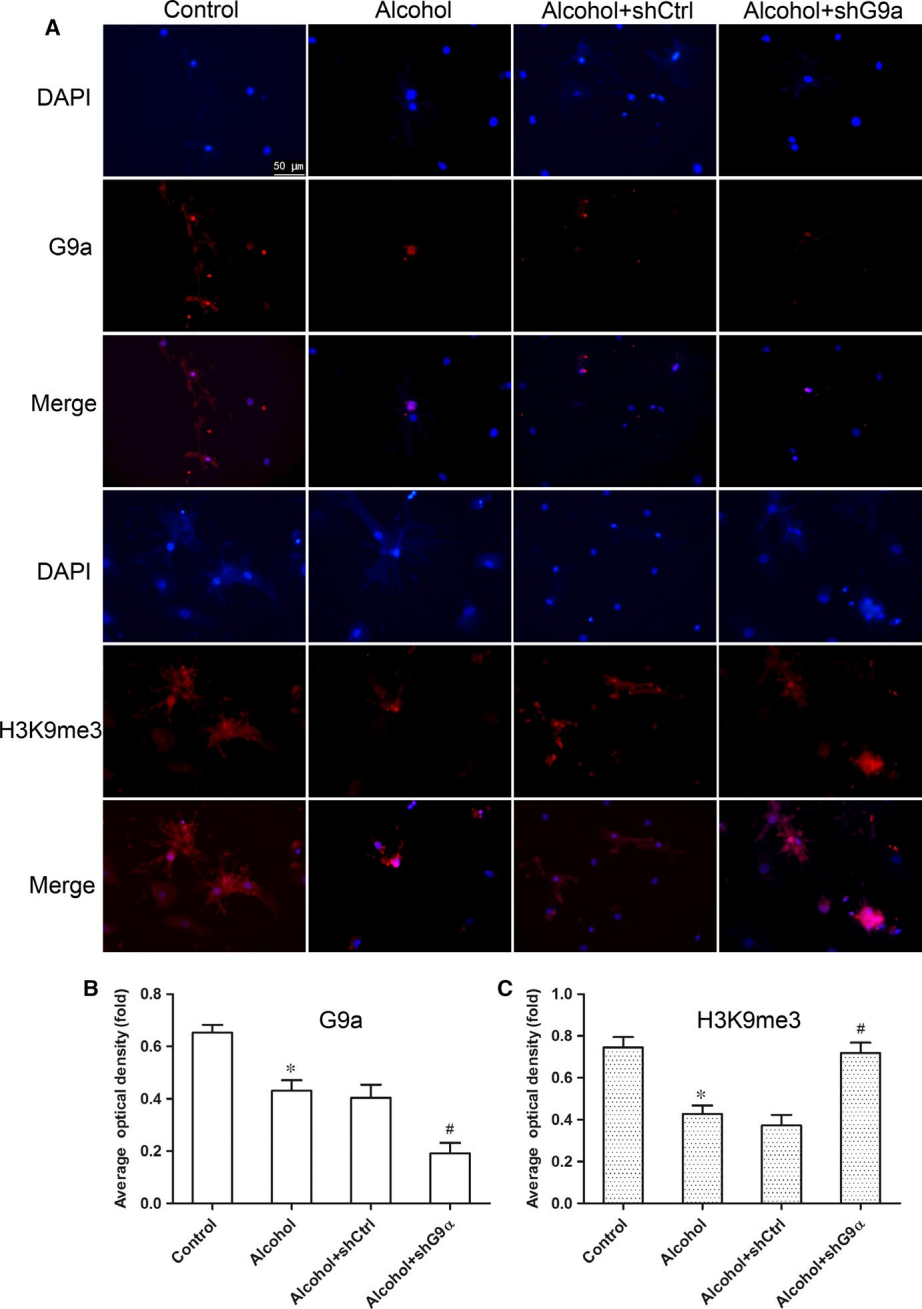
Effect of G9α knock‐down on the expression of G9α and H3K9me3 in alcohol‐exposed mouse myocardial cells. (A) G9α and H3K9me3 (red fluorescence) combined with DAPI (blue fluorescence) staining in myocardial cells exposed to four different conditions. The scale bars represent 50 μm. (B) Average optical density of G9α immunofluorescence in the four groups. (C) Average optical density of H3K9me3 immunofluorescence in the four groups. All results were representative of at least three independent experiments. *P < .05 vs the control group, #*P* < .05 vs the alcohol + shCtrl group (n = 3)

We analysed the cellular localization of G9α and H3K9me3 by immunofluorescence and found both proteins exclusively localized in the nucleus (Figure 4A). Moreover, myocyte exposure to alcohol inhibited G9α expression, which was also suppressed by G9α‐shRNA‐mediated knock‐down (Figure 4B). Moreover, H3K9me3 methylation was inhibited by alcohol, but higher H3K9me3 methylation was observed in alcohol‐exposed, G9α‐knock‐down cells compared to that in alcohol‐exposed, shCtrl‐transfected cells (Figure 4C). These data indicated that alcohol could decrease the methylation level of H3K9me3 by inhibiting the expression of G9α in primary cardiomyocytes.

### 3.5 Alcohol promotes the expression of cardiomyogenesis‐related genes by regulating G9α‐dependent histone H3K9me3 hypomethylation

**Figure 5:**
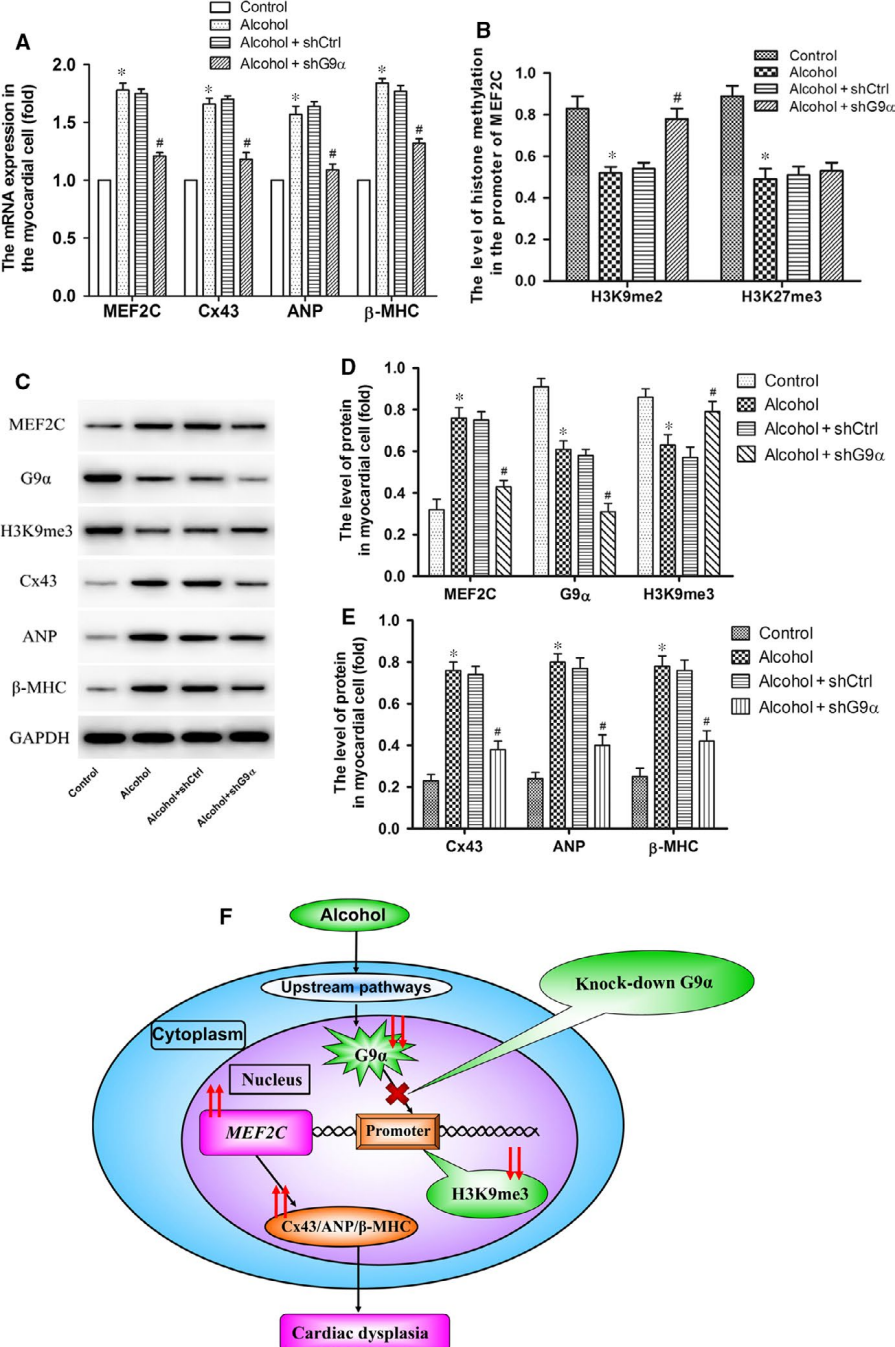
G9α‐dependent histone H3K9me3 hypomethylation promotes the overexpression of cardiomyogenesis‐related genes in alcohol‐exposed mouse myocardial cells. (A) RT‐PCR shows that alcohol induced the overexpression of *MEF2C* mRNA in myocardial cells exposed to alcohol, whereas G9α knock‐down prevented alcohol‐induced *MEF2C* overexpression in the same samples. Moreover, increased mRNA expression of *Cx43*, *ANP* and *β‐MHC* was observed after alcohol treatment, and G9α knock‐down prevented this effect. (B) The levels of histone H3K9me2 and H3K27me3 at the *MEF2C* promoter were significantly decreased after treatment with alcohol. G9α knock‐down prevented alcohol‐induced histone H3K9me2 underexpression, and the level of histone H3K27me3 was unchanged in shG9α‐transfected cells. (C and D) Western blotting shows that alcohol induced MEF2C protein overexpression in myocardial cells exposed to alcohol, whereas G9α knock‐down prevented this effect. Notably, a decrease in G9α and H3K9me3 protein expression was observed after treatment with alcohol, whereas G9α knock‐down abolished the alcohol‐induced G9α down‐regulation, and alcohol‐induced H3K9me3 hypermethylation was observed. (E) Western blotting shows that alcohol could not cause the overexpression of Cx43, ANP and β‐MHC in G9α‐knock‐down mouse myocardial cells. *P < .05 vs the control group, #*P* < .05 vs the shCtrl group (n = 6). (F) Illustration of the potential mechanism of alcohol‐induced abnormal expression of cardiomyogenesis‐related genes. G9α‐HMT‐mediated imbalance in histone H3K9me3 methylation plays a key regulatory role in the abnormal expression of cardiomyogenesis‐related genes induced by alcohol. When G9α is knocked out, alcohol cannot cause the overexpression of cardiomyogenesis‐related genes

RT‐PCR showed that the mRNA expression of *MEF2C*, *Cx43*, *ANP* and *β‐MHC* was higher in alcohol‐treated cells than in untreated cells; among alcohol‐treated cells, the expression of these genes was lower in shG9α‐transfected cells than in mock‐transfected cells (Figure 5A). ChIP‐qPCR assays indicated that the binding of histone H3K9me2 and H3K27me3 at the promoter region of *MEF2C* showed a significant decrease in alcohol‐exposed cells compared to that in controls. The level of histone H3K9me2 was significantly increased in shG9α‐transfected cells, whereas the level of histone H3K27me3 was unchanged in shG9α‐transfected cells (Figure 5B). Subsequently, Western blotting was used to evaluate the expression of G9α and H3K9me3. Both proteins exhibited a significantly lower expression in alcohol‐exposed cells than in controls. In addition, among alcohol‐exposed cells, the expression of G9α was further reduced in shG9α‐transfected cells compared to that in shCtrl‐transfected cells. Notably, H3K9me3 methylation was significantly increased in shG9α‐transfected cells. The protein expression of MEF2C, Cx43, ANP and β‐MHC was significantly higher in alcohol‐exposed cells than in control cells and was lower in alcohol‐treated/shG9α‐transfected cells than in alcohol‐treated/mock‐transfected cells (Figure 5C‐E).

## 4 DISCUSSION

Congenital heart disease is the most common congenital anomaly worldwide. Based on population data from China, it is estimated that 150 000 children with CHD are born every year. Congenital heart disease is the major cause of infant morbidity and death from birth defects.^16^ At present, treatment of CHD is poorly effective, also implying serious economic burden to families and society. Many studies have shown that alcohol consumption during pregnancy has increased worldwide.^17^, ^18^, ^19^ Notably, alcohol consumption during pregnancy has been found to cause CHD in foetuses.^20^, ^21^ However, the mechanistic link between alcohol and CHD is still largely unclear. Recently, numerous studies have shown that genetic and epigenetic mechanisms may play vital roles in CHD. Epigenetics represents a bridge between genetics and the environment. The major epigenetic events include histone methylation, acetylation and phosphorylation, DNA methylation, microRNA modifications and others. The interaction between cardiac transcription factors and histone methylation has a central role in cardiac development.^22^, ^23^ Our previous studies have further demonstrated that alcohol exposure during pregnancy leads to the abnormal expression of genes related to cardiac development and that the latter event depends on imbalanced histone acetylation. Unfortunately, inhibitors of histone acetylases can only partially reverse alcohol‐induced gene expression abnormalities.^12^ Therefore, we speculate that additional epigenetic mechanisms may be involved in this pathophysiological process. Thus, in this study, we explored the role of altered histone methylation in alcohol‐induced abnormal expression of cardiomyogenesis‐related genes. We hope that this study will provide a new theoretical foundation for the treatment of CHD caused by alcohol consumption during pregnancy.

Previous research has shown that both HMTs and histone demethyltransferases may be implicated in CHD pathogenesis.^24^, ^25^, ^26^ However, evidence of HMT involvement in alcohol‐induced CHD development is lacking. Our experiments showed significantly reduced HMT activity in the hearts of alcohol‐exposed foetal mice compared to that in control animals. However, it is known that HMTs include many subtypes.^27^, ^28^ We specifically examined G9α‐HMT, which is closely related to cardiac development and expressed in the heart. Western blotting analysis confirmed that the level of G9α was significantly lower in alcohol‐treated mice than in control animals and showed that alcohol caused histone H3K9me3 hypomethylation. It has been reported that the hypomethylation of H3K9me3 may be caused by the increase of the activity of KDM4A in hypertrophic cardiomyocytes.^29^ Noteworthily, the tendencies of the expression levels of both KDM4A and H3K9me3 were consistent with the data in our experiments. Moreover, the overexpression of cardiomyogenesis‐related genes was analysed in the foetal myocardial tissues of alcohol‐exposed mice. In addition, we evaluated the relationship between cardiac nuclear transcription factor *MEF2C* and downstream cardiac development‐linked genes. ChIP‐PCR indicated that *MEF2C* was involved in the regulation of *ANP*, *Cx43* and *β‐MHC* expression. These results suggested that altered G9α‐mediated histone methylation was involved in the abnormal expression of cardiomyogenesis‐related genes in alcohol‐exposed foetal mouse hearts. Hence, we speculate that G9α‐HMT may be crucial for alcohol‐induced overexpression of cardiomyogenesis‐related genes during prenatal exposure. To further confirm this hypothesis, primary cardiomyocytes of neonatal mice were cultured in vitro, and a lentiviral silencing system was used to knock‐down G9α expression in in vitro cultured myocardial cells.

Our data indicated that alcohol could significantly decrease the expression of G9α and H3K9me3 in the primary cardiomyocytes of neonatal mice. Furthermore, the results obtained from lentiviral vector‐mediated shRNA indicated down‐regulated G9α expression in cultured cardiomyocytes. However, among alcohol‐treated myocardial cells, H3K9me3 methylation was higher in shG9α‐transfected cells than in mock‐transfected cells. Thus, alcohol did not cause histone H3K9me3 hypomethylation in shG9α‐knock‐down mouse myocardial cells. In addition, the CoIP experiments indicated that G9α‐HMT interacted with H3K9me3 in these cells. Notably, our data indicated that alcohol could induce the overexpression of cardiomyogenesis‐related genes (*MEF2C*, *ANP*, *β‐MHC* and *Cx43*) in myocardial cells, whereas G9α knock‐down counteracted this effect. Overall, our data further support the important regulatory role of G9α‐HMT in the up‐regulation of cardiomyogenesis‐related genes induced by alcohol exposure (Figure 5F).

However, the possible role of upstream signalling pathways and whether other histone modifications in addition to methylation and acetylation are involved in alcohol‐induced cardiac dysplasia remain unclear. Further studies are urgently needed in the area of alcohol‐induced cardiac dysplasia.

## 5 CONCLUSIONS

Our study indicated that G9α‐HMT‐mediated histone H3K9me3 hypomethylation promotes the overexpression of cardiomyogenesis‐related genes in alcohol‐exposed foetal mouse hearts. The results implied that G9α‐HMT plays an important regulatory role in the overexpression of cardiomyogenesis‐related genes induced by alcohol exposure. Theoretically, G9α knock‐in approaches may provide a more clear‐cut demonstration of the role of G9α in alcohol‐induced CHD‐related events; nevertheless, G9α is difficult to knock‐in and the experimental conditions are relatively high. Thus, this study provides a theoretical foundation for the treatment of CHD caused by alcohol consumption during pregnancy.

## CONFLICT OF INTEREST

The authors confirm that there are no conflicts of interest.

## Data Availability

The data that support the findings of this study are openly available in [repository name, eg “figshare”] at http://doi.org/%5Bdoi], reference number [reference number].
