# Corrigendum

**DOI:** 10.1111/jcmm.17433

**Published:** 2022-07-06

In Bohui Peng et al,^1^ there is an image assembly error in the shCtrl group (Bright field) in Figure 3E. The correct figure is shown below. The authors confirm that all results and conclusions of this article remain unchanged.

**FIGURE 3:**
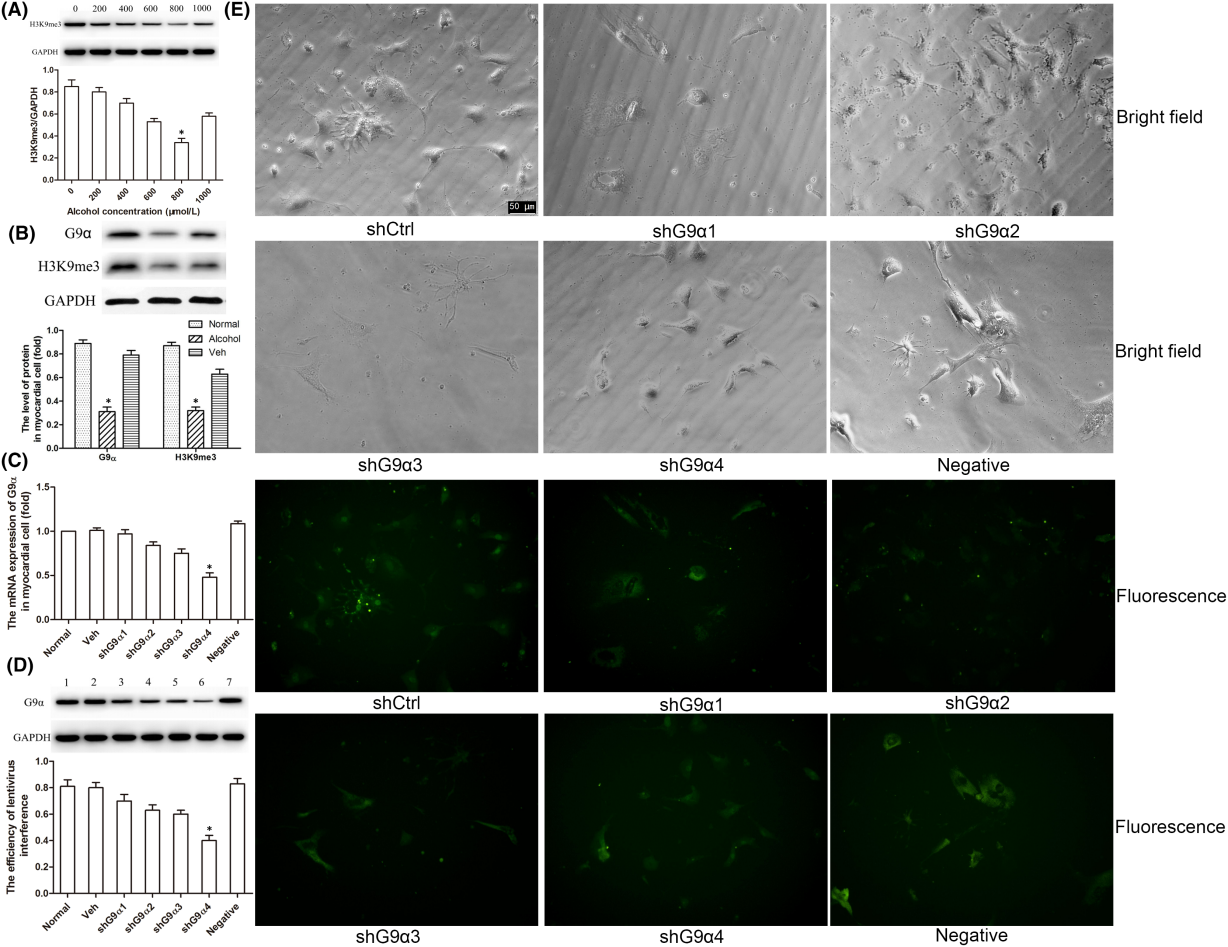
Lentivirus‐mediated shRNA transfection can efficiently and specifically knock down G9α gene expression in myocardial cells. (A) Different alcohol concentrations were used to determine the optimal exposure dose in primary cardiomyocytes of neonatal mice, and 800 μmol/L was selected based on the methylation level of H3K9me3. (B) The level of G9α and H3K9me3 showed a significant decrease in myocardial cells. (C) Four shG9α intervention sites were used to optimize the transfection efficiency, and shG9α4 was selected based on G9α mRNA expression in myocardial cells. (D and E) The efficiency of transfection with lentiviral vector containing G9α shRNA was analysed by Western blotting and immunofluorescence, respectively. The scale bars represent 50 μm. *p < 0.05 vs the control group (*n* = 6)
